# Survival of hypoxaemic patients treated with solar-powered oxygen in rural Somalia hospitals: a prospective, observational study

**DOI:** 10.1016/j.ebiom.2026.106196

**Published:** 2026-03-10

**Authors:** Mohamed M. Ali, Haron Ndwiga Njiru, Abdullah Al Azad, Md Shajib Hossain, Abdirashid Ali Asir, Iqbal Anwar, Guled Abdijalil Ali, Ali Haji Adam Abubakar, Sk Md Mamunur Rahman Malik, Abdinasir Yusuf Osman

**Affiliations:** a Department of Sexual, Reproductive, Maternal, Child, Adolescent Health and Ageing: Advancing Life Course Health and Reproduction (LHR), World Health Organization, Geneva, Switzerland; b World Health Organization, Mogadishu, Somalia; c South Health Campus, Alberta Health Services, Calgary, Alberta, Canada; d World Health Organization, Beirut, Lebanon; e Ministry of Health and Human Services, Federal Government of Somalia, Mogadishu, Somalia; f World Health Organization, Regional Office for the Eastern Mediterranean, Cairo, Egypt; g World Organization for Animal Health (WOAH) Collaborating Centre in Risk Analysis and Modelling, Food and Agriculture Organization of the United Nations (FAO) Reference Centre for Veterinary Epidemiology, Veterinary Epidemiology, Economics and Public Health, Department of Pathobiology and Population Sciences, Royal Veterinary College, London, UK

**Keywords:** Hypoxaemia, O_2_ concentrators, O_2_ delivery, Solar power, Somalia, Survival analysis

## Abstract

**Background:**

During the coronavirus disease 2019 (COVID-19) pandemic, solar-powered oxygen concentrator systems were established in rural hospitals of conflict-affected, hard-to-reach regions in Somalia to address acute gaps in oxygen access. We assessed the outcome of treatment of patients and risk factors for death among patients receiving oxygen for hypoxaemia in these settings in Somalia.

**Methods:**

We analysed data on all patients receiving medical oxygen for hypoxaemia in six rural hospitals in Somalia equipped with solar-powered oxygen concentrator systems during the pandemic. The endpoint of the analysis was death. We used the Kaplan–Meier survival analysis and the log-rank test to compare survival curves. We used Cox proportional hazard model to determine the predictors of death.

**Findings:**

We included 1460 patients (age from 1 day to 90 years) with hypoxaemia (peripheral blood saturation level <90%) treated with solar-powered oxygen concentrator systems in six rural hospitals in Somalia between February 2021 and December 2023. There were 103 deaths, 22 of which occurred within 24 h of detection of hypoxaemia in the hospital. The Kaplan–Meier survival analysis showed the cumulative hazard of death at 1.5% (95% CI 1.1–2.3%) during the first 24 h of admission, 11.9% (95% CI 8.1–17.3%) on day 7 post-admission conditional on surviving the first 24 h. Patients aged 1–11 months had a significantly increased hazard of death (aHR 7.71 [95% CI 2.54–23.41]) while those admitted with birth asphyxia were 4 times more likely to die compared to patients with pneumonia (aHR 3.83 [95% CI 1.12–13.06]). Improved patient outcomes were associated with higher oxygen saturation levels on admission. One unit increase in SpO_2_ level was associated with nearly 10% decrease in the hazard ratio of death (aHR 0.9 [95% CI 0.88–0.92]).

**Interpretation:**

The availability of solar-powered oxygen concentrator systems can be a reliable solution to address oxygen insecurity in fragile, vulnerable health systems and conflict-affected countries, and can be associated with improved patient outcomes. Early detection and rapid initiation of oxygen therapy for hypoxaemic illness can support improved survival in patients of all ages, and with different conditions, if health care facilities without reliable electricity are fitted with solar-powered oxygen concentrator systems.

**Funding:**

The authors have received no funding for this study.

**Research in context**

**Evidence before this study**

The therapeutic use of oxygen in healthcare facilities has been limited in countries affected by conflict and humanitarian crises owing to lack of availability. The COVID-19 pandemic exacerbated medical oxygen shortages in these settings. Previous studies have shown that solar energy can be used to generate oxygen from ambient air using concentrators and can be used to reduce gaps in oxygen access in resource-constrained settings. These studies provide evidence of the effectiveness of solar-powered oxygen concentrator systems in saving lives in peripheral health facilities without any access to reliable electricity. All these studies have focused on the value of oxygen in correcting hypoxaemia in children and for diseases that mainly affect neonates, infants, and children.

**Added value of this study**

This study is on survival analysis of 1460 patients with hypoxaemia admitted to six rural hospitals situated in conflict zones in Somalia. These hospitals are government-funded general hospitals in hard-to-reach areas without any reliable source of electricity. The primary endpoint of our analysis was death during the first 24 h of admission due to hypoxaemic conditions and to the end of observation period, which was day 11 following the admission (when the last death was reported). Patients of any age (between 1 day and 90 years) and with any disease condition causing hypoxaemia were included in the analysis. In Kaplan–Meier survival analysis, the estimated cumulative hazard of death for any hypoxaemic patient with SpO_2_ below 90% was 1.5% (95% CI 1.1–2.3%) during the first 24 h and 28.4% (95% CI 15.3–48.8%) at the end of the observation among those who survived day one. Improved patient outcomes were associated with higher SpO_2_ level on admission for all patients irrespective of age or medical condition. Our study included a large number of patients across all age groups and with all medical conditions, including COVID-19. The survival benefit of using solar-powered oxygen concentrator systems in hypoxaemic conditions is examined in a conflict zone during an ongoing pandemic.

**Implications of all the available evidence**

Our study has shown that the solar-powered oxygen concentrator systems can be successfully deployed in healthcare facilities of conflict zones for treatment of hypoxaemia in all ages and with multiple clinical conditions. Implementing solar-powered oxygen concentrator systems in active conflict zones could fill critical gaps in oxygen access where there may not be any reliable source of electricity. These systems can provide an uninterrupted and reliable supply of oxygen for various acute and chronic medical conditions and could contribute to improved clinical outcomes for patients with hypoxaemia. Our finding that higher SpO_2_ level on admission was associated with improved outcomes in hypoxaemic patients implies that early detection of hypoxaemia and availability of solar-powered oxygen concentrator systems should be prioritised in all healthcare facilities, even in active conflict zones where people are underserved and do not have access to reliable oxygen availability.

## Introduction

Medical oxygen is an essential lifesaving medicine needed for many medical and surgical conditions for which no substitute exists. Despite its inclusion in the World Health Organization's (WHO's) list of essential medicine in 2012,^1^ and wide recognition that it is a fundamental component of any country's healthcare system, poor and inadequate access to medical oxygen or unreliable oxygen services has been a persistent issue in most healthcare facilities of the low- and middle-income countries (LMICs).^2^ Lack of sustainable financing for oxygen services and data on oxygen needs, inadequate oxygen service capacity in health facilities, and absence of any national plan to improve oxygen delivery and integrate it into essential health services are the main reasons for lack of availability of medical oxygen in various countries despite its critical importance in healthcare. Securing sustainable investment to establish and maintain reliable oxygen services for healthcare in LMICs has also been challenging due to cost, or operational and other logistical challenges.^3^ Even in affluent nations, disparities in access to oxygen was a major health system problem during the COVID-19 pandemic. In addition to lack of evidence or data gaps regarding existing service coverage and oxygen needs, rapid surge in demand quickly depleted oxygen supplies within healthcare facilities. The logistic bottlenecks in getting oxygen from the industrial providers to the healthcare facilities, shortages of equipment needed to provide oxygen to patients posed significant challenges to optimising oxygen availability in these rich countries as well. There was no coordinated global actions or synthesis of knowledge among countries, partners, private investors, the business community and donors on reliable, affordable and adaptable solutions that could build and maintain sustainable oxygen systems in every country, rich or poor.

Although medical oxygen has long been used to treat a wide range of infectious diseases, acute medical and chronic conditions, and for safe surgery, and emergency, maternal and neonatal care, the COVID-19 pandemic exacerbated the shortages of medical oxygen in healthcare settings, possibly causing excess mortality.^2^ The Lancet Global Health Commission on medical oxygen security highlighted that large global inequities exist in medical oxygen access. The commission estimated that nearly 70% of people in LMICs who need oxygen for acute medical or surgical conditions do not have access to it in healthcare settings.^4^ An important recommendation of the Commission was to synthesise evidence for sustainably scaling up access to oxygen in LMICs as an effective intervention for addressing oxygen insecurity.

Somalia is among the most fragile and conflict-affected states in the world today.^5^ The country has suffered from armed conflict, civil war, political instability, and repeated climate-related emergencies for the past three decades. In 2022, Somalia's Human Development Index (HDI) was 0.380, the lowest of 193 countries and territories,^6^ and the country's economic growth, averaged between 2019 and 2023, showed a negative real gross domestic product per capita growth rate.^7^ The economic fragility and protracted conflicts have severely weakened the healthcare system in the country. The universal health coverage index for Somalia is 27 out of 100 against a global average of 60.3,^8^ and there are fewer than one skilled health worker per 1000 people in a population of nearly 17 million.^9^ The country has one of the highest maternal, newborns and under five mortality rate in the world (Supplementary Materials, pp. 1–2).^10^

At the start of the COVID-19 pandemic, Somalia lacked medical oxygen for treatment of severely ill patients in both public and private sector hospitals. A previous study showed improved survival of severely ill COVID-19 patients following treatment with only oxygen over other interventions.^11^ Most hospitals had no high-grade medical oxygen available with reports suggesting that only 26% of health facilities in the country had access to medical oxygen during the pandemic.^12^ When the demand for oxygen surged during the COVID-19 pandemic, WHO assisted the government of Somalia to rapidly increase oxygen access across the country. The strategy aimed at ensuring affordable and sustainable medical oxygen for everyone (Supplementary Materials, pp. 3–4). The biggest challenge was to ensure oxygen availability at health facilities in active conflict zones which serve poor, rural and marginalised population. Prolonged civil war and armed conflict had severely damaged health services in these areas and many health facilities were functioning with minimum resources without reliable access to electricity. Six small-sized secondary level care hospitals situated in these active conflict zones were equipped with solar-powered oxygen concentrator systems during the pandemic to ensure people in these difficult settings can access medical oxygen closer to their homes. In 2021, the first of these systems was established in a rural hospital, approximately 600 km from the capital city of Somalia. The success of this first solar-powered oxygen concentrator system in terms of health benefit, cost, compliance and client satisfaction has been previously described.^13^ Inspired by this success and with support from WHO and other partners, five additional rural hospitals were also equipped with solar-powered oxygen concentrator systems between 2022 and 2023.

There are few data from conflict-affected countries on the benefits of solar-powered oxygen concentrator systems for the treatment of hypoxaemia in people of all ages and with different medical conditions. There is evidence that availability of solar-powered oxygen concentrator systems was associated with reduced mortality in resource-constrained settings, but such evidence has primarily emerged in paediatric populations and for diseases that mainly affect neonates, infants, and children.^14^, ^15^, ^16^, ^17^ As hypoxaemia is a key predictor of in-hospital mortality and affects people of all ages with many different conditions, we aimed to address the knowledge gap on the benefits of solar-powered oxygen delivery in treating hypoxaemia across all ages and multiple conditions in a fragile and vulnerable setting. By integrating solar-powered oxygen delivery into routine healthcare practice in the conflict zones of Somalia, we wanted to evaluate if the availability of these oxygen services was associated with improved health outcome in patients of all ages and with any medical condition. Our objective was to assess patient outcomes and identify the risk factors associated with death in patients treated with solar-powered oxygen for hypoxaemia in secondary level hospitals of conflict zones in Somalia.

## Methods

### Study design

This was a prospective, observational study conducted between February 2021 and December 2023 in six rural hospitals in Somalia which were equipped with solar-powered oxygen concentrator systems for delivery of medical oxygen to COVID-19 patients.

### Patients

We included patients of any age and with any medical condition if they were acutely ill, had difficulty breathing, had hypoxaemia (peripheral oxygen saturation level below 90%) and required hospitalisation in any of the six hospitals. 1460 patients were enrolled from 12 February 2021 to 27 December 2023 from these six sites (Supplementary Materials p. 5, Table S1).

### Study settings

The six secondary-level hospitals are small district hospitals serving between 250,000 and 1 million people and with a bed capacity ranging from 30 to 50 beds. Characteristics of the study sites are available in the Supplementary Materials (pp. 6–7, Tables S2 and S3). The annual outpatient visits for the six hospitals vary from 6908 to more than 46,000 and in-patient admissions vary from 497 to over 10,000 a year. Between 98 and 1200 basic surgeries are performed in these hospitals annually. Road travel time from the hospitals to Mogadishu ranges from 12 h to 2 days. Access to the hospitals by air is restricted owing to insecurity and armed conflict. These hospitals are poorly resourced, do not have any reliable source of electricity, are financed mostly by local clans or humanitarian agencies and had no reliable source of oxygen before the solar-powered concentrator systems were installed. The solar-powered oxygen concentrator systems were installed in the emergency department, in operating theatre and in general wards.

### Procedures

As part of standard patient care, the indication for oxygen therapy was determined for each patient by clinical examination, history taking and screening for hypoxaemia using pulse oximetry upon admission. Based on clinical condition judged by the attending physician or nurse, patients whose SpO_2_ level was <90% on admission and who had breathing difficulty were hospitalised and put on oxygen immediately irrespective of the reasons for hypoxaemia. The patients were given oxygen either continuously or intermittently, depending on the severity of their condition and their response to oxygen treatment. The oxygen flow rates were titrated and adjusted as necessary using the concentrator's flowmeter and the SpO_2_ level was continuously monitored with pulse oximetry to guide therapy. A standard protocol for oxygen delivery for hypoxaemia was developed and used in all six hospitals. Patients were regularly monitored for improvement in oxygen saturation level until they were discharged without any disability after being weaned off oxygen, died, or were referred to another hospital. A standard procedure for weaning off oxygen therapy was followed to safely reduce or discontinue the treatment. At night, patients were closely monitored by the attending nurse and/or on-call physician at the admitting hospital. All patients also received standard care for their underlying disease (Supplementary Materials, pp. 8–10). At least two qualified nurses in each hospital recorded patient data prospectively including: date of admission, age, sex, SpO_2_ level on admission and on discharge, clinical diagnosis, final outcome (discharged alive, including patients remaining in hospital up to the last day of the study, transferred, or died) and date of final outcome. Information on sex was self-reported by the study participants. The data for individual patients were transcribed every month onto an Excel spreadsheet with unique patient identification numbers (Microsoft Corp., Redmond, WA, USA). A focal point at each hospital (another senior care provider) independently verified the information for completeness and accuracy at the end of every week. The monthly data received from each hospital at the WHO country office were compiled in a master database for analysis.

### Outcomes

Our primary outcome for analysis was time to in-hospital death. This was measured from admission to hospital and administration of oxygen to time of death. Patients who were discharged without disability, transferred to another facility or were still in hospital at the end of the observation period (27 December 2023) were censored on the day of discharge, transfer, or last day of observation, respectively.

### Covariates

In addition to time to death, we included the patient's age category (1 day–<1 month, 1–11 months, 1–<5 years, and 5–90 years) and sex (male/female). We also recorded two risk factors: indication for receiving oxygen therapy i.e. clinical diagnosis (birth asphyxia; pneumonia; COVID-19/other respiratory diseases; newborn disorder; and other diseases including injuries); and SpO_2_ level (30–60%, 61–75%, and 76–89%) The detailed reasons for admission for receiving oxygen therapy for hypoxaemia and scheme for grouping of these diseases/indications for receiving oxygen therapy are available in the Supplementary Materials (pp. 11–13, Table S4).

### Statistics

We used percentages to summarise categorical variables and means and standard deviations for continuous variables. We used ANOVA to assess the within covariates differences in SpO_2_ on admission, and at the end of the observation, controlling for the hospital and used the paired t-test to assess the within-patient differences in SpO_2_ between the two time points. We used the Kaplan–Meier method to estimate survival probabilities (with 95% confidence intervals [CI]) during the first 24 h of detection of hypoxaemia, and from day two to the time last censored (still in hospital) patient, conditional on surviving the first 24 h, stratified by covariates. We used the log-rank test to assess the significance of differences in survival probabilities of patients with different covariates. We fitted Cox proportional hazard models with SpO_2_ as a continuous variable and added a frailty term to control for between-hospital heterogeneity. We reported the adjusted hazard ratios (aHR) of death during hospitalisation during the first 24 h, after day one conditional on surviving the first 24 h, and for the entire observation period. The assumption of the Cox proportional hazard model was examined for age and sex and for the two risk factors—indication for receiving oxygen therapy and SpO_2_ levels. We used Stata Version 19.0 (StataCorp LLC, College Station, TX, USA) for all analyses.

### Ethics

The study was approved by the National IRB, Ministry of Health & Human Services, Galmudug State of Somalia, Somali Federal Republic (Ref: MoH/GMS/DGO-2021–2026). With respect to human participants, the requirements for written informed consent were waived because these solar-powered oxygen concentrator systems were used for life-saving purposes during an ongoing pandemic and the waiver was approved by the IRB. All data were collected as part of clinical care and standard hospital data collection procedure, where all individual records were anonymised.

### Role of funders

No funding was received for this study. The authors are solely responsible for study design, data collection, data analysis, data interpretation, and writing of the manuscript.

## Results

Between 12 February 2021 and 27 December 2023, 1460 patients received medical oxygen from solar-powered oxygen concentrator systems for hypoxaemia in six hospitals where these systems were installed. There were 12 patients who were still in the hospitals at the end of the study period receiving oxygen for hypoxaemia and were censored. About half of the patients (46.6%) were treated with oxygen in Hanano hospital, followed by 28.8% in Kismayo hospital and the rest in other four hospitals (Table 1). The greatest proportion of patients were aged 1 day–<1 month (41.0%) and 21.6% were between 1 year and <5 years. The median age of the patients was 7 months (IQR <1 month–4 years). There were slightly more male patients (51.5%) than females (48.5%). SpO_2_ levels on admission ranged from 30% to 89% with a mean of 69.4% (standard deviation 13.96%), the SpO_2_ was further disaggregated by covariates (Supplementary Materials pp. 14–17, Figure S1A–D). Pneumonia (34.9%) and birth asphyxia (32.0%) were the commonest reasons for receiving oxygen for hypoxaemia (SpO_2_ <90% on admission). The patient's characteristics were also stratified by the survival status after the first 24 h of admission. Those who died in the first 24 h were from two hospitals and male patients are nearly as twice as female patients who died in the first 24 h of admission.

**Table 1:** Characteristics of patients who received oxygen for treatment of hypoxaemia including those who had died by day 1 and those who were alive 24 h after admission.

	All Patients		Survival status end of day one			
	n	(%)	Died		Survived	
			n	(%)	n	(%)
**Health facilities**						
Bay	75	(5.1)	0	(0.0)	75	(5.4)
Bossaso	159	(10.9)	0	(0.0)	159	(11.3)
Hanano	681	(46.6)	21	(36.2)	660	(47.1)
Hawladag	80	(5.5)	0	(0.0)	80	(5.7)
Jowhar	45	(3.1)	0	(0.0)	45	(3.2)
Kismayo	420	(28.8)	37	(63.8)	383	(27.3)
**Age groups**						
Less than a month	598	(41.0)	18	(31.0)	580	(41.4)
1–11 months	188	(12.9)	8	(13.8)	180	(12.8)
1–4 years	315	(21.6)	9	(15.5)	306	(21.8)
5+ years	359	(24.6)	23	(39.7)	336	(24.0)
**Sex**						
Male	752	(51.5)	38	(65.5)	714	(50.9)
Female	708	(48.5)	20	(34.5)	688	(49.1)
**Indication for Oxygen therapy**						
Birth asphyxia	467	(32.0)	13	(22.4)	454	(32.4)
Pneumonia	509	(34.9)	4	(6.9)	505	(36.0)
COVID19/Other Respiratory Diseases	122	(8.4)	10	(17.2)	112	(8.0)
Newborn Disorder	102	(7.0)	9	(15.5)	93	(6.6)
Others including Injuries	260	(17.8)	22	(37.9)	238	(17.0)
**Oxygen saturation level on admission (SpO_2_)**						
30–60%	435	(29.8)	26	(44.8)	409	(29.2)
61–75%	474	(32.5)	10	(17.2)	464	(33.1)
76–89%	551	(37.7)	22	(37.9)	529	(37.7)
**Total**	**1460**	**(100.0)**	58	**(100.0)**	1402	**(100.0)**

Overall, 1316 patients (90.1%) were discharged from hospital without any disability after receiving oxygen therapy successfully (Table 2) and 41 patients (2.80%) were either transferred to other health facilities or remained in the hospital at the end of the observation period for treatment of their medical conditions. There were only 103 deaths (7.05%) among hypoxaemic patients who received medical oxygen during the entire study period including 22 that occurred within 24 h of detection of hypoxaemia in the hospital. The mean duration of hospitalisation for those who were discharged without any disability (after successfully correcting SpO_2_ level for hypoxaemia) was 2.26 days (standard deviation 1.59).

**Table 2:** The mean and (SD) of peripheral oxygen saturation level (SpO_2_) by covariates, on admission and at the end of observation period, measured by pulse oximetry.

	On admission				At the end of observation				Differences		
	n	Mean	SD	P-value^c^	n^a^	Mean	SD	P-value^c^	Mean	SE	P-value^d^
**Health facilities**				<0.0001				<0.0001			
Bay	75	69.0	(7.4)		73	94.5	(12.3)		25.7	(1.68)	<0.0001
Bossaso	159	65.9	(7.3)		159	88.8	(26.7)		22.9	(1.98)	<0.0001
Hanano	681	64.1	(14.1)		680	98.6	(5.5)		34.5	(0.57)	<0.0001
Hawladag	80	69.6	(9.7)		79	98.3	(5.5)		28.7	(1.23)	<0.0001
Jowhar	45	62.9	(6.2)		44	99.3	(0.6)		36.3	(0.96)	<0.0001
Kismayo	420	80.1	(11.5)		420	89.7	(26.1)		9.5	(1.01)	<0.0001
**Age groups**				<0.0001				0.0021			
Less than a month	598	67.0	(14.1)		597	92.8	(21.5)		25.9	(0.95)	<0.0001
1–11 months	188	73.7	(12.6)		186	94.2	(18.1)		20.5	(1.44)	<0.0001
1–4 years	315	72.8	(12.9)		314	96.9	(11.3)		24.1	(0.93)	<0.0001
5+ years	359	68.4	(14.2)		358	96.4	(14.9)		28.0	(1.03)	<0.0001
**Sex**				0.8292				0.2936			
Male	752	69.5	(13.7)		751	94.3	(18.9)		24.8	(0.76)	<0.0001
Female	708	69.3	(14.2)		704	95.3	(16.5)		25.9	(0.77)	<0.0001
**Indication for Oxygen therapy**				<0.0001				0.0002			
Birth asphyxia	467	66.3	(14.6)		466	93.6	(20.3)		27.3	(1.05)	<0.0001
Pneumonia	509	73.0	(13.4)		507	97.4	(9.8)		24.4	(0.71)	<0.0001
COVID19/Other Respiratory Diseases	122	70.7	(14.6)		122	95.8	(15.4)		25.1	(1.60)	<0.0001
Newborn Disorder	102	68.6	(11.7)		102	90.9	(24.0)		22.3	(2.34)	<0.0001
Others including Injuries	260	67.8	(12.8)		258	92.6	(22.0)		24.8	(1.54)	<0.0001
**Final disposition of patients**^b^				<0.0001				<0.0001			
Discharged without disability	1316	70.1	(14.0)		1315	98.6	(14.0)		28.6	(0.39)	<0.0001
Died	103	61.0	(10.9)		102	43.9	(10.9)		−17.0	(3.89)	<0.0001
Referred to another facility/or still in hospital	41	70.6	(14.3)		38	96.9	(14.8)		26.4	(2.87)	<0.0001
**Total**	1460	69.4	(14.0)		1455	94.7	(17.8)		25.3	(0.54)	<0.0001

a 5 missing observation: 1 died, 1 measure not taken on discharge, 3 were still inpatient at the end of the study.

b The mean hospital duration of those who were discharged without any disability was 2.26 days (Standard Deviation 1.59).

c ANOVA.

d Paired t-test single-side H_a_: mean > 0.

The mean SpO_2_ level of all the 1460 patients (Table 2) on admission (upon detection of hypoxaemia) was 69.4% (standard deviation 14.0%) while the mean SpO_2_ of 1455 patients whose SpO_2_ level was available at the end of the observation period was 94.7% (standard deviation 17.8%). The SpO_2_ on admission and at the end vary significantly with all covariates except age.

The SpO_2_ of all the 1316 patients, who survived and were discharged without any disability, improved from a mean of 70.1% (standard deviation 14.0%) at the time of detection of hypoxaemia to a mean of 98.6% (standard deviation 14.8%) at the time they were weaned off oxygen. This represented a significant mean increase of 28.6% (standard error 0.39; paired t-test = 74.07, P < 0.0001) (Table 2).

**Fig. 1:**
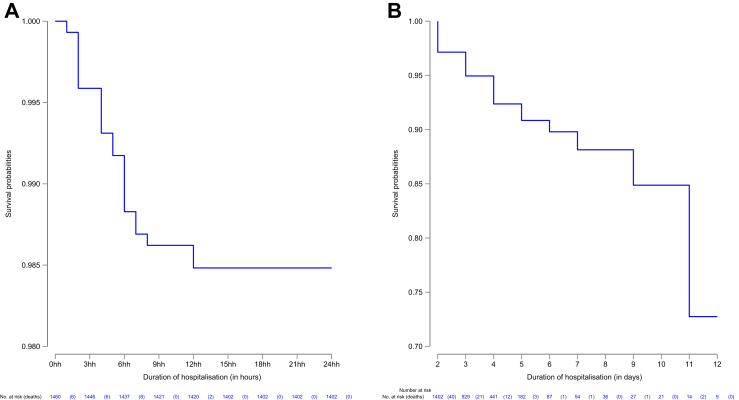
Kaplan–Meier survival estimates for 1460 hypoxaemic patients who received oxygen during the first 24 h (Panel A) and after the first 24 h conditional on surviving the first 24 h (Panel B).

The Kaplan–Meier survival curves are shown in Fig. 1. By the end of the first 24 h (Panel A), the cumulative hazard of death was 1.5% (95% CI 1.1–2.3%). Among the 1402 patients who survived the first 24 h and still hospitalised (Panel B), the cumulative hazard of death was 11.9% (95% CI 8.1–17.3%) on day 7 post-admission conditional on surviving the first 24 h. Among the remaining 36 patients under observation after day 7, one died on day nine and two on day 11.

**Fig. 2:**
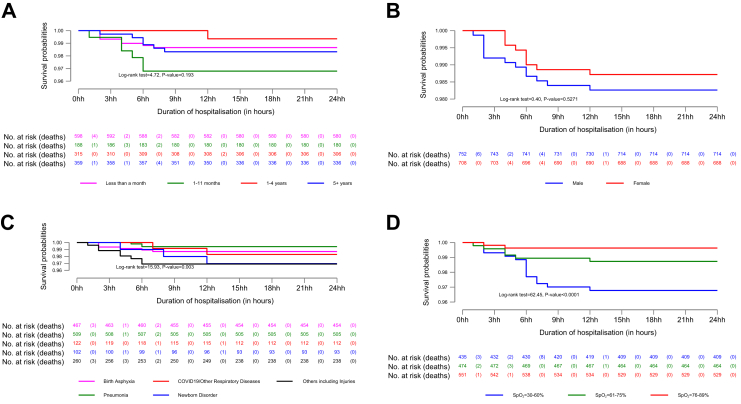
Stratified Kaplan–Meier survival probabilities during the first 24 h of hypoxaemic patients, by age (Panel A), sex (Panel B), indication for receiving oxygen therapy (Panel C) and SpO_2_ level on admission (Panel D).

**Fig. 3:**
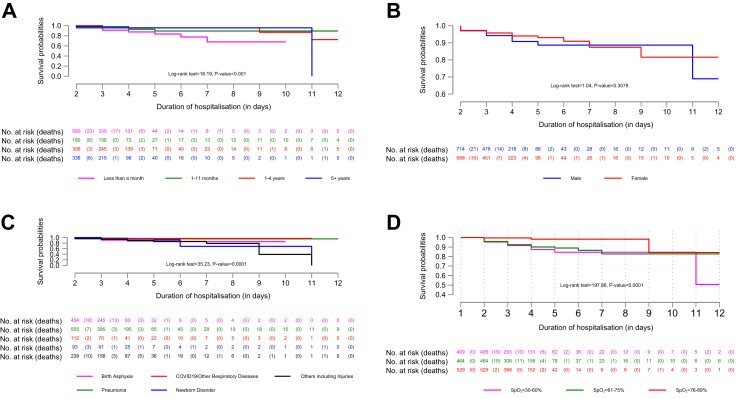
Stratified Kaplan–Meier survival probabilities after the first 24 h conditional on surviving the first 24 h of hypoxaemic patients, by age (Panel A), sex (Panel B), indication for receiving oxygen therapy (Panel C) and SpO_2_ level on admission (Panel D).

The survival probabilities during the first 24 h (Fig. 2) and after day one (Fig. 3) were further stratified by covariates. All covariates, except sex, were significantly associated with hazard of death in both time periods. The Kaplan–Meier curves show that during the first 24 h, the hazard of death was higher in patients aged 1–11 months, patients with hypoxaemia due to newborn disorders or other diseases including injuries, and patients whose SpO_2_ level on admission was 30–60%. Among patients who survived the first 24 h, the hazard of death was higher in patients aged 1 day–<1 month but remained unchanged for other two covariates. The difference in survival was statistically significant between age group (log-rank test = 16.19, P = 0.001), indication for receiving oxygen therapy and SpO_2_ level (log-rank test = 35.23, P < 0.0001).

Before fitting the Cox models, we assessed the proportional hazard assumption for SpO_2_ level on admission by hospital, by age group, sex, hospital and indication for receiving oxygen therapy separately, and adjusting for hospitals (Supplementary Materials pp. 18–21, Figure S2A–D). The results confirmed that the assumption held. In the Cox model (Table 3), we estimated the adjusted HR of death during the first 24 h, after day one conditional on surviving the first 24 h, and for the whole study period. After controlling for age, sex, indication for receiving oxygen therapy and SpO_2_ level on admission, during the first 24 h of admission, the hazard of death among hypoxaemic patients was nearly eight times higher in the age group 1–11 months than those <1 month (aHR 7.71 [95% CI 2.54–23.41]). Birth asphyxia was associated with nearly four times higher hazard of death than pneumonia (aHR 3.83 [95% CI 1.12–13.06]), as were other diseases including injuries. The SpO_2_ level on admission was strongly associated with increased hazard of death. One unit increase in SpO_2_ level was associated with a decrease in the HR of nearly 10%, (aHR 0.90 [95% CI 0.88–0.92]) during the first 24 h, after the first 24 h and during the whole study period.

**Table 3:** Adjusted hazard ratios (aHR) of death in patients receiving medical oxygen for hypoxaemia.

Variable/characteristics	First 24 h			After the first day			Entire period		
	aHR	95% CI	P-value	aHR	95% CI	P-value	aHR	95% CI	P-value
**Age group**									
Less than a month	1.00			1.00			1.00		
1–11 months	**7.71**	**(2.54**–**23.41)**	<0.0001	1.62	(0.62–4.24)	0.323	**2.86**	**(1.24**–**6.61)**	0.014
1–4 years	**2.46**	**(0.69**–**8.72)**	0.164	1.27	(0.49–3.30)	0.617	1.73	(0.73–4.07)	0.210
5+ years	2.88	(1.02–8.16)	0.047	1.01	(0.42–2.48)	0.974	1.45	(0.67–3.13)	0.343
**Sex**									
Female	1.00			1.00			1.00		
Male	1.21	(0.72–2.03)	0.473	1.09	(0.68–1.73)	0.730	1.08	(0.72–1.63)	0.700
**Indication for Oxygen therapy**									
Pneumonia	1.00			1.00			1.00		
Birth asphyxia	**3.83**	**(1.12**–**13.06)**	0.032	**2.88**	**(1.09**–**7.62)**	0.033	**3.44**	**(1.42**–**8.38)**	0.006
COVID19/Other Respiratory Diseases	1.52	(0.39–5.96)	0.548	0.71	(0.16–3.12)	0.646	1.13	(0.34–3.71)	0.846
Newborn Disorder	3.19	(0.84–12.13)	0.089	1.85	(0.60–5.72)	0.287	**2.77**	**(1.04**–**7.40)**	0.042
Others including Injuries	**3.08**	**(1.27**–**7.52)**	0.013	**2.89**	**(1.30**–**6.40)**	0.009	**3.27**	**(1.64**–**6.52)**	0.001
**Oxygen saturation level on admission (SpO_2_)**	**0.90**	**(0.88**–**0.92)**	<0.0001	**0.91**	**(0.90**–**0.93)**	<0.0001	**0.91**	**(0.89**–**0.92)**	<0.0001
Unobserved heterogeneity: thetah (SE)	5.19	(3.67)		1.97	(1.01)		2.12	(1.07)	
LR test of theta = 0	168.92		<0.0001	158.80		<0.0001	227.73		<0.0001

aHR: adjusted Hazard Ratio, aHR = 1.00 indicates the reference category, 95% CI: 95% Confidence Interval. Bold values indicate statistically significance (P < 0.05).

Birth asphyxia as well as other diseases including injuries were also associated with higher hazard of death in patients who survived the first 24 h in hospital. Over the whole study period, birth asphyxia, newborn disorders, other diseases including injuries, and age 1–11 months were associated with a higher hazard of death. The corresponding unadjusted HRs are available in the (Supplementary Table S5, pp. 22).

## Discussion

This study demonstrated that the solar-powered oxygen concentrator systems were successfully deployed in six rural hospitals of Somalia even during an ongoing pandemic. Overall, 1460 patients received medical oxygen from solar-powered oxygen concentrator systems for hypoxaemia in these six hospitals which were all situated in an active conflict zone. In this study, we have assessed the outcome of all 1460 patients.

We found improved outcomes in patients with hypoxaemia of all ages and with many medical conditions, including COVID-19, following treatment with medical oxygen from solar-powered oxygen concentrator systems. We also observed improved treatment outcome in patients with higher peripheral oxygen saturation level on admission.

Overall, 1316 patients (90.1%) were successfully treated and discharged without any disability from the hospital within a mean hospitalisation duration of 2.26 (SD = 1.59) days. During the entire study period, 103 deaths (7.05%) occurred, of which 22 deaths were reported within 24 h of admission (upon detection of hypoxaemia). No death was reported beyond day 11 of our observation period. We have found that the overall cumulative hazard of deaths (at the end of the observation period) was 28.4% (95% CI 15.3–48.8%), 1.5% (95% CI 1.1–2.3%), during the first 24 h, and 11.9% (95% CI 8.1–17.3%) on day 7 post-admission conditional on surviving the first 24 h in all age groups and medical conditions.

The results of our survival analysis show that children aged 1 day–<5 years had a higher HR of death. Birth asphyxia and other diseases including injuries had a higher HR of death during the first 24 h, while birth asphyxia, newborn disorders and other diseases including injuries had higher HR during the whole study period. While the probability of death was similar for males and females, we have observed that probability of survival was greater with higher levels of SpO_2_ on admission. One unit increase in SpO_2_ level on admission was associated with a nearly 10% decrease in HR (p˂0.0001).

Our findings support the increasing body of evidence that the solar-powered oxygen concentrator systems can improve patient outcomes in resource-constrained settings where access to uninterrupted and reliable electricity impedes safe delivery of oxygen.^13^, ^14^, ^15^, ^16^, ^17^, ^18^ Although these solar-powered oxygen concentrator systems were originally installed to provide oxygen to COVID-19 patients, all acutely ill hypoxaemic patients in all ages and with many medical conditions benefitted from this oxygen delivery.

Many studies conducted in resource-constrained settings have also found improved patient outcomes following treatment via solar-powered oxygen concentrator systems. For example, a study in the Democratic Republic of the Congo found a 50% decrease in 30-day mortality in children <5 years with hypoxaemia from respiratory illness after implementation of solar-powered oxygen concentrator systems in two rural hospitals.^16^ In Papua New Guinea, the establishment of solar-powered oxygen concentrator systems in remote hospitals supported by improvement in the quality of care was associated with a 40% reduction in overall paediatric mortality and a >50% reduction in mortality from pneumonia among children from birth to 13 years.^19^ A study in two hospitals in Uganda^15^ compared a solar-powered oxygen concentrator systems with standard delivery using compressed oxygen cylinders in children <13 years with hypoxaemia. In-hospital mortality was similar for the two groups (risk difference 4.6% [95% CI –7.8 to 17.0%]), as was the increase in peripheral blood oxygen saturation and time to wean off oxygen. Another study in a resource-constrained paediatric hospital in Uganda on using solar-powered oxygen concentrator systems for pneumonia in children <5 years reported six deaths (21%) among 28 children with hypoxaemia.^14^ A cluster randomised controlled trial on the outcome of treatment with solar-powered oxygen concentrator systems for children with hypoxaemia in 20 rural Ugandan hospitals found that use of solar-powered oxygen resulted in a relative risk reduction of 48.7% (95% CI 8.5–71.5%) in 48-h mortality.^3^

Our findings have important implications for Somalia and other conflict-affected countries with similar health systems and barriers to improved healthcare, especially for hard-to-reach areas. Somalia has one of the highest neonatal and under-5 death rates globally.^20^ Although precise data are not available on the causes of these high rates given the absence of a civil registration system, a multicentre hospital-based study^21^ found that the commonest causes of deaths in neonates were asphyxia (21%), premature birth (13%), and neonatal sepsis (9%). Somalia also has a high burden of childhood pneumonia,^10^^,^^22^ with about 21% of deaths in children <5 years attributed to pneumonia.^23^ Hypoxaemia is a major complication of these child health conditions and is a strong predictor of death.^24^ However, improved treatment outcome can be seen in children with hypoxaemia if oxygen is available at points-of-care.^25^ Thus, integrating reliable oxygen services such as solar-powered oxygen concentrator systems in routine healthcare practice at facility level and simultaneously scaling up other evidence-based health interventions such as antenatal care, family planning, basic emergency obstetric and newborn care, childhood immunisation, treatment of common childhood illnesses, skilled birth attendance and targeting populations living in high-risk setting can be a game-changer for Somalia and other resource-constrained countries. Engaging community health workers and expanding the use of outreach services to bring healthcare closer to populations will be fundamental for delivering and achieving high healthcare coverage. This was seen during the COVID-19 pandemic. Somalia achieved high coverage for COVID-19 vaccines in a challenging environment through engaging local community and the community health workers. An integrated package of services was delivered through organising health outreach closer to where the people lived.^26^ These health interventions, if implemented at scale with good-quality healthcare, and ensuring availability of antibiotics at the primary care level, can reduce the risk of death from childhood pneumonia and other causes of neonatal and infant mortality in high-risk settings.^25^ As Somalia recovers from the pandemic, this investment in solar-powered oxygen concentrator systems may unlock an opportunity to equitably scale up other evidence-based interventions and achieve gains in children's and women's health.

These solar-powered oxygen concentrator systems have been rapidly deployed in remote locations during the pandemic in Somalia, are cheap, affordable, scalable, and do not need skilled personnel for repair/maintenance.^13^ These systems are adaptable and can be integrated into routine health-care practice at primary, secondary and tertiary level care. As these systems use renewable energy, their widespread use will help reduce carbon emissions in healthcare. Economic evaluation of solar-powered oxygen concentrator systems has shown their cost–effectiveness compared with grid-powered oxygen concentrators or fuel generator-powered concentrators.^27^ The use of solar energy to power oxygen concentrators can ensure an uninterrupted supply of oxygen in peripheral healthcare settings, allowing a longer and more consistent supply of oxygen therapy for those requiring it.^16^^,^^17^

We consider that the wider use of pulse oximetry by frontline health workers at primary-care level can improve early recognition of severe hypoxaemia and trigger early initiation of oxygen therapy. If peripheral health-care centres are equipped with solar-powered oxygen concentrator systems and health workers are trained on the use of pulse oximetry for measuring SpO_2_ and know when to initiate oxygen therapy, this may improve patient's outcome. This is consistent with the recommendation of the Lancet Global Health Commission on medical oxygen security^4^ and other studies suggesting wider use of pulse oximetry in the community settings to detect hypoxaemia, and guide decisions for oxygen therapy.^3^^,^^24^^,^^28^^,^^29^ Solar-powered oxygen concentrator systems also benefit patients with severe injuries and acute medical and surgical conditions. We found an elevated hazard of death associated with hypoxaemia in these medical conditions, with many patients benefitting from early initiation of oxygen therapy. In countries experiencing armed conflict and violence, many patients present with life-threatening injuries and trauma and need immediate oxygen support. If peripheral health facilities of these countries, situated in off-grid and inaccessible locations, are equipped with solar-powered oxygen concentrator systems and pulse oximetry, this could facilitate early treatment with reliable oxygen and contribute to improved patient's outcome for these severely injured patients with hypoxaemia. Transfer of critically injured patients from these hard-to-reach areas to other higher facilities outside may be challenging or impossible owing to insecurity, inaccessibility and poor road infrastructure.

As this was an observational study, the causal inferences on the association between patient outcomes and improvement in SpO_2_ level cannot be confidently drawn. Such associations have been established in randomised control trials (RCTs) and other pre- and post-intervention studies on solar-powered oxygen concentrator systems conducted in the past.^3^^,^^15^, ^16^, ^17^^,^^19^ For this study, we were unable to assess the correlation between the patient outcomes and other variables. We conducted this study during an ongoing pandemic, in a very fragile, weak and debilitated hospital environment where the solar-powered oxygen concentrator systems were deployed on an emergency basis as a life-saving measure. The ethical concerns and other logistical difficulties to conduct any experimental research in an under-resourced environment while simultaneously providing life-saving services during an ongoing pandemic have been previously described by other researchers as well.^30^^,^^31^ Despite certain limitations, our study included large numbers of patients across all age groups and medical conditions, including COVID-19 where we have examined the benefits of using solar-powered oxygen concentrator systems for acute hypoxaemic conditions in a conflict zone. Experienced nurses who were trained on the use of pulse oximetry and oxygen concentrators collected the data prospectively each day. They knew that records were being kept for donor reporting but were unaware that patient mortality was being studied. The quality of the information collected was verified by another skilled care provider, therefore, recording bias was kept to minimum. Furthermore, mortality (the primary outcome) was studied at different time intervals: at 24 h, and after 24 h (for patients surviving the first 24 h and up to the last death reported). This was a prospective, multi-centre study, and we examined the association of SpO_2_ levels with improved patient outcomes. Our study findings could be used as a decision guide for early initiation of oxygen therapy for patients whose SpO_2_ levels on admission are found to be critically low. Our statistical analysis plan was adequately powered to adjust for the association between SpO_2_ and patient outcomes. Finally, we conducted Cox regression model analysis with frailty test to adjust for the effect of SpO_2_ on the collected risk factors after examining proportional hazard assumption. We also conducted all prespecified sensitivity analyses, which confirmed our main findings. Additionally, except for SARS-COV-2 infections which were laboratory-confirmed using rapid diagnostic tests, the other diseases presenting with hypoxaemia were diagnosed clinically as no laboratory or radiological tests were available in the study hospitals to confirm diagnosis.

During the COVID-19 pandemic, medical oxygen insecurity was a significant challenge to Somalia's health care system. The implementation of solar-powered oxygen concentrator systems filled critical gaps in oxygen access in conflict zones and improved patient outcomes. We also observed improved treatment outcome in patients with higher peripheral oxygen saturation level on admission. As such, we recommend the use of pulse oximetry at the community and primary care level for early detection of hypoxaemic illness in the community, timely referral to health centres equipped with solar-powered oxygen concentrator systems and early initiation of oxygen therapy. We advocate for scaling up the use of solar-powered oxygen concentrator systems, implementing a standardised procedure for provision of oxygen to patients with a hypoxaemic illness caused by any medical condition and close monitoring of the therapy. Integrating solar-powered oxygen concentrator systems into routine healthcare practice in fragile and conflict-affected countries can be transformative. If these interventions are accompanied by standard data collection on the use of solar-powered oxygen and patient outcomes through the integrated disease surveillance system such as DHIS 2, it will help assess the effectiveness of these systems in fragile settings and guide decisions for future investments on such oxygen systems in insecure and inaccessible areas. The solar energy used to power oxygen concentrator systems can be used to provide renewable green energy for entire health facilities in areas without reliable electricity. Electrifying healthcare facilities has been linked to improvements in service delivery and quality of care for various routine, emergency and critical care procedures, especially for newborns and women in many resource-constrained countries.^32^ The use of renewable energy in healthcare of fragile countries such as Somalia will help build a sustainable climate-resilient health system without increasing carbon emissions, which will eventually contribute to better health and well-being for the disadvantaged populations of marginalised settings.

## Contributors

MMA designed the study, did the data analysis, participated in writing and reviewing the paper. HNN accessed, managed and verified the data and also participated in the writing, reviewing and editing the paper. AAI supervised the data collection and participated in reviewing and editing the paper. IA participated in the data analysis, reviewing and editing the paper. SH, AAA, GAA and AHAA participated in reviewing and editing the paper. SMMRM participated in designing the study and wrote the first draft of the manuscript. AYO participated in writing the subsequent drafts of the paper and also reviewed and revised the final paper. Access and verification of underlying data: MMA and SMMRM. All authors read and approved the final version of the manuscript before submission for publication.

## Data sharing statement

Data will be made available upon reasonable request to the corresponding author mentioning the proposed use of the data. Requests for data sharing should be addressed to nasir@moh.gov.so.

## Declaration of interests

The authors declare that they have no competing interests. The authors alone are responsible for the views expressed in this publication, and they do not necessarily represent the decisions, policy, or views of WHO.
