# Supplementary material for: Survival of hypoxaemic patients treated with solar-powered oxygen in rural Somalia hospitals: a prospective, observational study
Source: eBioMedicine. 2026 Mar 10;126:106196. doi: 10.1016/j.ebiom.2026.106196 (PMC12995464; doi:10.1016/j.ebiom.2026.106196)
Supplement: Supplementary Figures and Tables [file mmc1.pdf]

<u>**Supplementary materials (appendix)**</u>

Survival of hypoxaemic patients treated with solar-powered oxygen in rural Somalia hospitals

Table of Contents

Supplemental Methods: Description of the study context, Somalia 1

Supplemental Information: strategy adopted to address oxygen access gap in Somalia during the COVID-19 pandemic 3

Table S1. Enrolment and discharge dates of 1460 hypoxaemic patients (SpO_2 less than 90%) included in the study, by hospital 5

Table S2. Characteristics of study sites (rural hospitals) 6

Table S3. Annual workload of study sites (rural hospitals) 7

Supplementary methods: description of interventions 8

Table S4. Scheme for grouping of reasons for admission of 1,460 patients with hypoxaemia (indications for receiving oxygen) 11

Figure S1 A-D. Distribution of SpO_2 on admission, by selected covariates 14

Figure S2 A-D. Assessing the proportional-hazard assumption for each covariate separately, and adjusting for hospitals 18

Table S5. Crude hazard ratios of death during first 24 hours, after first day and the entire period 22

Reference 23

Supplemental Methods: Description of the study context, Somalia

Somalia (population 18,358,615 as of 2023)[1] is an East African country in sub-Saharan Africa and is ranked among the most fragile and conflict-affected states in the world.[2] The country has suffered from armed conflict, civil war, political instability, and repeated climate-related emergencies for the past three decades. In 2022, Somalia's Human Development Index (HDI) was 0.380, the lowest of 193 countries and territories,[3] and the country's economic growth, averaged between 2019 and 2023, showed a negative real gross domestic product per capita growth rate.[4] The economic fragility and protracted conflicts have severely weakened the healthcare system in the country. The universal health coverage index for Somalia is 27 out of 100 against a global average of 60.3,[5] and there is fewer than one skilled health worker per 1,000 people in the country.[6] The country has one of the highest newborns and under five mortality rate in the world with 36.15 per 1000 live births and 142.9 per 1000 live births respectively in 2022.[1] The life expectancy at birth for Somalia was 54 years in 2021.[1] The leading causes of deaths (both for males and females) in the country include lower respiratory tract infections, diarrhoeal diseases, preterm birth complications, maternal conditions, birth asphyxia and birth trauma.[1]

Distribution of health centres, district hospitals, and tertiary care centres

Four different levels of health facilities provide essential health services in Somalia. The lowest level is called the primary health units (PHU) which are also called health centres (HC) or mother and child health (MCH) centres. These centres offer outpatient services for basic healthcare, including maternity and child health care but do not provide any emergency care services. The next higher level comprises of district hospitals, situated at district level but are predominantly in rural settings, regional hospitals are situated in the administrative regions and national hospitals are situated at the state capital cities. In the recent report of Harmonised Health Facility Assessment conducted in 2023[8], there are 1, 219 operational health facilities in the country. Of these, 67% are primary health units, 15% are private clinics and 15% are hospitals which include all types of hospitals (district hospital, regional hospitals and national hospitals). This means that the country has 0.69 health facilities of any type per 10,000 people. The government manages only 52% of all the 1, 219 operational health facilities while 25% are managed by non-governmental organization

and 20% are managed by private-for-profit organization. The services between all levels of care remain fragmented and uncoordinated in the absence of a properly functioning referral system.

As the health services are provided through a mix of public, private, and humanitarian actors with minimal control, regulation or coordination, this has led to inequitable access and duplication of efforts in some areas, and complete absence of health services in other areas. Beginning 2015, the private sector emerged as the main provider of health-care services in the country which also suffered from poor quality, high cost and inadequate coverage. There has been no new investment on the existing or new health infrastructure in the country since 1991 and the country 's internal spending on health care provision is one of the lowest in the world [8].These deeper structural failures have made the health services virtually dysfunctional leaving less than 30% of the country's population with access to basic health care [9] and the deep-rooted and systemic health inequalities and disparities have converged to create a deadly landscape for women, children and other vulnerable populations in the country.

Somalia does not have a national power grid and healthcare facilities either use a fossil fuel energy source or power from a private source to run their biomedical equipment. These energy-intensive sources are aften unaffordable to healthcare facilities for delivery of care. Even when electricity is available, frequent power interruptions and outages occur making the use of oxygen concentrators for delivery of medical oxygen challenging. The latest report of Harmonised Health Facility Assessment (national report, October 2024) indicates that only 41% health facilities in Somalia reported having electricity from any source.[7]

Supplemental Information: strategy adopted to address oxygen access gap in Somalia during the COVID-19 pandemic

During May–June of 2020, when the first wave of the COVID-19 pandemic hit Somalia, the fragility of the country's health system was clearly exposed: it struggled to manage patients with severe symptoms of COVID-19. People were needlessly dying because of the lack of available medical oxygen. As the situation continued to worsen, the availability of oxygen in the country was assessed jointly by the government and WHO. A survey conducted by WHO in 2022 showed that only 26% of health facilities surveyed had at least one oxygen source, while 4% had oxygen concentrators and 22% had access to oxygen cylinders.[10] This situation led the government and WHO to develop a data-driven strategy for scaling up oxygen availability in Somalia using a phased approach. Based on the data, a three-pronged strategy was implemented to scale up the availability of high-grade medical oxygen throughout the country from 2021 to 2023.[10]

1. **Equipping primary care with oxygen.** In the first phase, WHO provided 376 peripheral primary health care centres with oxygen concentrators and distributed pulse oximeters to more than 3000 community health workers.
2. **Installing solar-powered oxygen delivery systems (concentrators).** As only one in four health facilities in Somalia had access to the power grid or an uninterrupted supply of electricity, WHO, with support from Grand Challenges Canada, installed the first solar-powered oxygen concentrators in Hanano hospital in Galmudug, one of the most insecure areas of Somalia. Since then and after seeing the success of this solar-powered O_2 delivery system, WHO assisted the government to equip additional five district hospitals with solar-powered oxygen concentrator systems.
3. **Procuring pressure swing adsorption (PSA) plants**. In the third and final phase, WHO has installed containerized PSA plants in three large, specialized hospitals. Each plant (with two containers) has the capacity to refill 100 to 150 40-litre oxygen cylinders every day and simultaneously provide high-grade medical oxygen to 25 intensive care beds. At least 1800 40-litre oxygen cylinders are being refilled every month from the PSA plants serving more than 1000 patients a month in need of medical oxygen.

This strategy has pushed the country to move towards achieving self-sufficiency in medical oxygen in the country. Though total demand for medical oxygen in the country is yet to be estimated but it is planned in the near future. In the recent Harmonized Health Facility Assessment (2022-2023), it was found out that nearly 518 (87.2%) of the 594 health facilities assessed in the accessible areas had at-least one source of reliable oxygen services. [7]

Table S1. Enrolment and discharge dates of 1460 hypoxaemic patients (SpO_2 less than 90%) included in the study, by hospital

Hospital	First admission date	Last admission date	Last discharge date	Number recruited
Bay	19-Apr-22	22-Dec-23	26-Dec-23	75
Bossaso	01-May-23	24-Dec-23	26-Dec-23	159
Hanano	12-Feb-21	26-Dec-23	27-Dec-23	681
Hawladag	06-Mar-23	25-Dec-23	25-Dec-23	80
Jowhar	06-Jun-23	17-Dec-23	24-Dec-23	45
Kismayo	22-Apr-22	24-Dec-23	27-Dec-23	420
**Total**				**1,460**

Table S2. Characteristics of study sites (rural hospitals)

Name of hospital	State	Level of care	Date solar-powered oxygen concentrators system installed	Catchment population (estimated)	Power cuts
Bosaso hospital	Puntland	District hospital (secondary)	15 April 2023	350,000	Frequent
Bay regional hospital	Southwest	District hospital (secondary)	31 March 2022	1.048.615	Frequent
Johar regional hospital	Hirshabelle	District hospital (secondary)	1 June 2023	600,000	Frequent
Howladag hospital	Southwest	District hospital (secondary)	20 February 2023	250,000	Frequent
Hanano hospital	Galmudug	District hospital (secondary)	08 February 2021	780,000	Frequent
Kismayo hospital	Jubaland state	District hospital (secondary)	31 March 2022	400,000	Frequent

Table S3. Annual workload of study sites (rural hospitals)

Health facility	Catchment population	Annual workload*				
		EU visits (all)	EU visits (U5)	OPD visits	Inpatient admissions	Emergency operations
Bosaso Hospital	350,000	7890	679	6,908	987	168
Bay Regional Hospital	1,048,615	11,791	958	13,104	1,245	681
Hanano Hospital	780,000	15,478	1,278	46,890	497	877
Hawladag Hospital	250,000	9089	905	27090	670	98
Jowhar Regional Hospital	600,000	6,720	2,880	34,140	6,960	167
Kismayo General Hospital	400,000	6,917	706	25,508	10,029	1,296
**Average**	**571,436**	**9,648**	**1,234**	**25,606**	**3,398**	**548**

*Between 2022-2023. EU: emergency unit; OPD: out-patient; U5 (under 5 years)

All these six hospitals were equipped with solar-powered oxygen concentrator system which consisted of the following commercially available products-a 16 X 380W solar panels (JASolar, China), a charge controller (SmartSolar MPPT 250/60-Tr, Victron Energy BV, Almere Haven, The Netherlands); a bank of 16X 200Ah batteries (Ritar Gel Deep Cycle Batteries, Hunan, China); a DC-AC current inverter (Victron Energy BV); and between 6 to 10 300W oxygen concentrators (model 525 KS, DeVilbiss Healthcare LLC, Somerset, PA, USA). In addition, each of these hospitals received hand-held pulse oximetry, nasal prong for delivery of high-flow oxygen and training of healthcare workers on the use of pulse oximetry for measuring oxygen saturation level and delivery of high-grade medical oxygen through the solar-powered oxygen concentrators. The solar-powered system uses photovoltaic cells to collect solar energy, which is stored in a battery bank and used to power oxygen concentrators for the production of medical grade oxygen from ambient air. The battery banks enable uninterrupted flow of oxygen through the night and on cloudy days. These hospitals were selected for equipping with solar-powered oxygen concentrator systems because people living in the surrounding areas could not travel to other places to seek health care for critical illness owing to insecurity in the areas.

Supplementary methods: description of interventions

Each of the six hospitals, where the study was conducted, received between 6–10 oxygen concentrators and 6 oxygen cylinders as backup. The capacity of each oxygen concentrator was 10 L which were WHO pre-qualified equipment and well calibrated. The concentrators were fitted with solar power and were utilized in the emergency department, inpatient wards, and operating theatre of the hospital. The district hospitals in Somalia generally do not have separate units such as Pulmonology, Gynaecology or Intensive Care Units (ICUs). Instead, these services are integrated within existing departments of the hospital including in emergency or general wards where oxygen therapy is provided as needed.

As part of standard patient care, the indication for oxygen therapy was determined for each patient by clinical examination, history taking and screening for hypoxaemia using pulse oximetry upon admission. Based on clinical condition judged by the attending physician or nurse, patients whose SpO_2 level was <90% on admission and who had breathing difficulty were hospitalised and put on oxygen immediately irrespective of the reasons for hypoxaemia. The patients were given oxygen either continuously or intermittently, depending on the severity of their condition and their response to oxygen treatment. The oxygen was delivered via tubing connected to the concentrator's outlet, and the mode of delivery depended on the patient's clinical needs either by Nasal Cannula (requiring low to moderate oxygen flow (1–6 L/min), or Simple Face Mask (when oxygen requirements (5–10 L/min) or Non-Rebreather Mask (for higher oxygen concentration for severely hypoxaemic patients (10–15 L/min). The oxygen flow rate was adjusted using the concentrator's flowmeter, and oxygen saturation (SpO_2) level was continuously monitored with pulse oximetry to guide therapy. Humidification was, sometimes, added through a water humidifier bottle attached to the concentrator outlet, especially for higher flow rates, to prevent airway dryness.

WHO prequalified pulse oximetry was used throughout the study to ensure accuracy and reliability, and the device was calibrated properly. To minimize inter-device and observer variability standard procedure were put in place. The HCWs involved in the use of pulse oximetry were trained in proper device use, sensor placement, and interpretation of readings. Consistent device performance checks and repeat measurements, when necessary, further ensured that the

recorded oxygen saturation values were highly accurate and dependable. A standard protocol for oxygen delivery for hypoxaemia was developed in Somali language and used in all six hospitals. Patients were regularly monitored for improvement in oxygen saturation level until they were discharged without any disability after being weaned off oxygen, died, or were referred to another hospital. A standard procedure for weaning off oxygen therapy was also followed to safely reduce or discontinue the treatment. At night, patients were closely monitored by the attending nurse and/or on-call physician at the admitting hospital. All patients also received standard care for their underlying disease. At least two qualified nurses in each hospital recorded patients' data prospectively including: date of admission, age, sex, SpO_2 level on admission and on discharge, clinical diagnosis, final outcome (discharged alive, including patients remaining in hospital up to the last day of the study, transferred, or died) and date of final outcome.

These six hospitals were not well equipped for providing high-end emergency and critical care services owing to its frail and fragile conditions. The health care workers (HCWs) of these six hospitals were trained by team of WHO (including subject matter experts from WHO headquarters) before the installation of solar-powered oxygen concentrator system. The HCWs were specially permitted by the non-state actors (NSAs- Al-Shabab militant armed group) controlling the access of these areas to come to Mogadishu and receive hands-on training as they (NSAs) considered this initiative as lifesaving for these access-constrained local community people. The HCWs, comprised of physicians and nurses of the hospitals and were trained on the use of oxygen concentrators, maintenance and upkeep of concentrator, detection of hypoxaemia using the pulse oximetry and monitoring appropriate oxygen flow rates in paediatric and adult patients. WHO also organized periodic virtual continuing medical education (CME) sessions with these trained health workers during the entire study period. Additionally, a standard procedure for weaning from O2 therapy was developed in Somali language which was followed by all HCWs to determine if oxygen could be safely reduced or discontinued. A WHO Biomedical Engineer was assigned for monitoring the photovoltaic (PV) panel output, and battery state of charge in real using the Victron Remote Management online tool (Victron Energy BV). This tool provided measurements of these parameters at one-minute intervals. The oxygen content of the output stream from the concentrators was measured at the beginning and end of oxygen therapy using an oxygen analyser (Guangdong Pigeon Medical Apparatus Co, Canto, China) for all patients who

received oxygen for hypoxaemia. This was closely monitored virtually by WHO Biomedical Engineer throughout the entire study period.

Table S4. Scheme for grouping of reasons for admission of 1,460 patients with hypoxaemia (indications for receiving oxygen)

Final category	Group name	Diagnosis	Number
Birth asphyxia	*Birth asphyxia*	Birth asphyxia	467
Pneumonia	*Pneumonia*	Pneumonia	509
COVID-19	*COVID-19*	COVID-19	27
Newborn Disorder			
	Anaemia		
		Anaemia	27
		Leukaemia, unspecified.	1
	Newborn disorder		
		Cephalohaematoma due to birth injury	1
		Developmental anomalies, unspecified	1
		Hydrocephalus, unspecified	1
		Infections in the puerperium, unspecified	5
		Neonatal aspiration of meconium	47
		Neonatal seizures	8
		Preterm newborn	11
Injury			
	Injury		
		Fractures involving multiple body regions	5
		Gunshot wound of abdomen associated with armed conflict	23
		Gunshot wound of chest associated with armed conflict	7
		Injuries to the head	9
		Injury of femoral artery	1
		Injury of unspecified body regions	16
		Injury to the neck	1
		Intracranial injury	2
	Road Traffic Injury		
		Land transport road traffic injury	8
		Unintentional land transport road traffic injury event	1
Other Respiratory Disease			
	Lower Respiratory Track Disease		
		Asthma	54
		Chronic obstructive pulmonary disease	2
		Haemothorax	8
		Lower respiratory tract disease, unspecified	1
		Pulmonary oedema	1

	Spontaneous tension pneumothorax	3
	Unspecified asthma with status asthamiticus	1
Upper Respiratory Track Disease		
	Acute bronchiolitis	2
	Acute respiratory distress syndrome	19
	Acute upper respiratory infection	1
	Pleural effusion	3
Others		
Cardiovascular Disease		
	Acute myocardial infarction	1
	Chronic ischaemic heart disease, unspecified	2
	Congestive heart failure	7
	Hypertensive crisis	10
Disease of the Digestive System		
	Peptic ulcer	
	Cerebral ischaemic stroke	13
	Cerebral Palsy, unspecified	2
	Cerebral ischaemia	1
	Status epilecticus	4
	Kidney failure, unspecified	1
	Nephrotic syndrome	2
	Neurofibromatoses	1
Metabolic Disease		
	Diabetic acidosis	34
Other Communicable Disease		
	Bacterial meningitis, unspecified	1
	Infectious meningitis, unspecified	3
	Malaria without parasitological confirmation	5
	Measles	1
	Tetanus	5
	Tuberculosis	5
Others		
	Anaphylaxis, unspecified	1
	Bacteraemia	1
	Dehydration	1
	Drowning or neonatal submersion	1
	Fluid overload	1
	Harmful effects of biological substance	3
	Hypovolaemic shock	15
	Incisional hernia	1
	Shock, unspecified	1
	Tuberculosis, unspecified	1
	Ventricular septal defect	1

Pregnancy-related disorder	
Eclampsia	9
Sepsis	
Sepsis	53

Figure S1 A: SpO_2 on admission, by Hospital

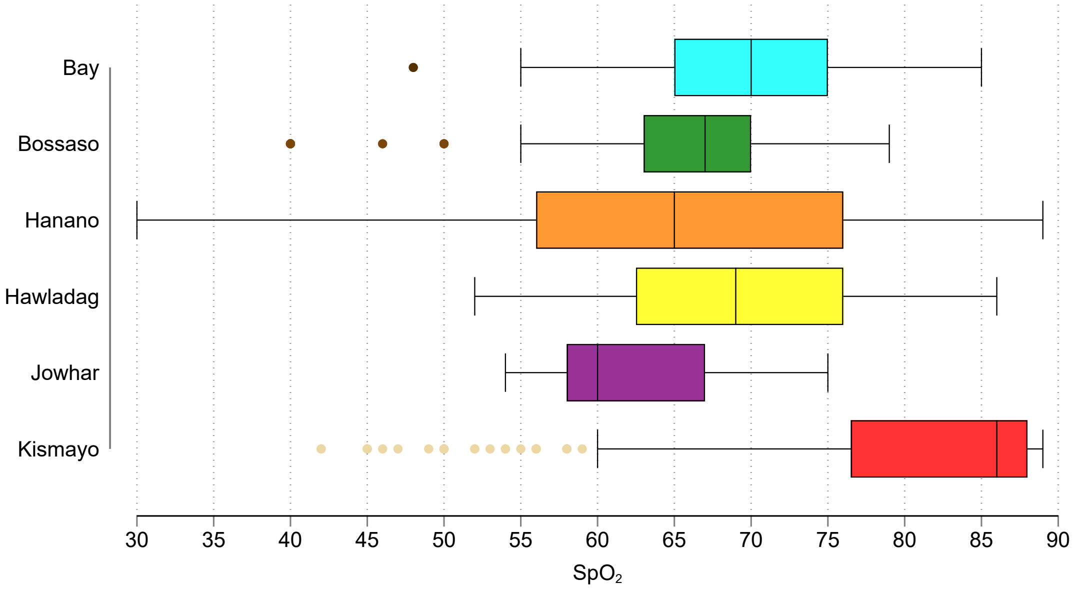

Figure S1 B: SpO_2 on admission, by Age group

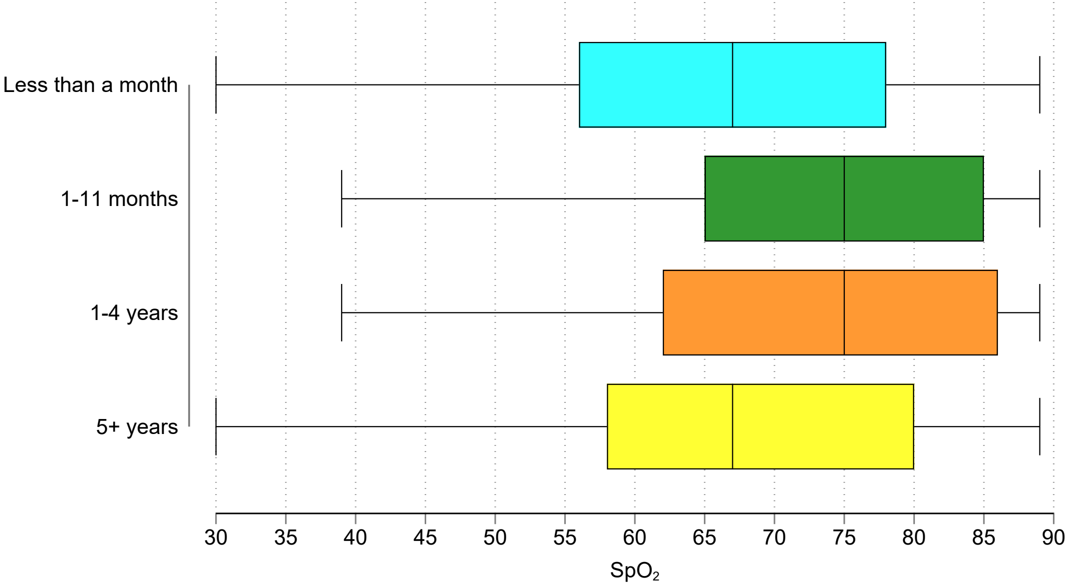

Figure S1 C: SpO_2 on admission, by Sex

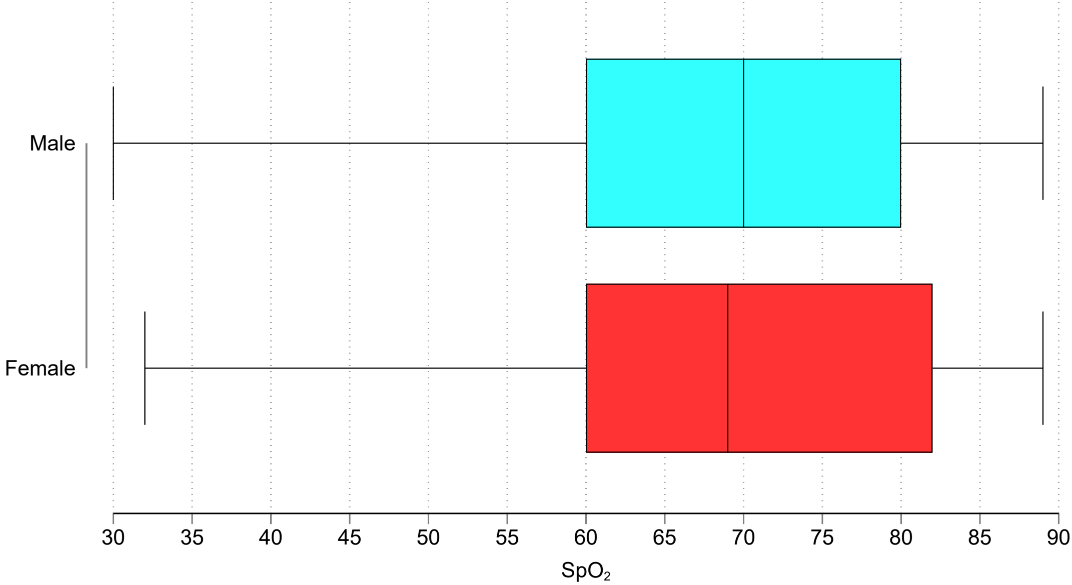

Figure S1 D: SpO_2 on admission, by Indication for Oxygen therapy

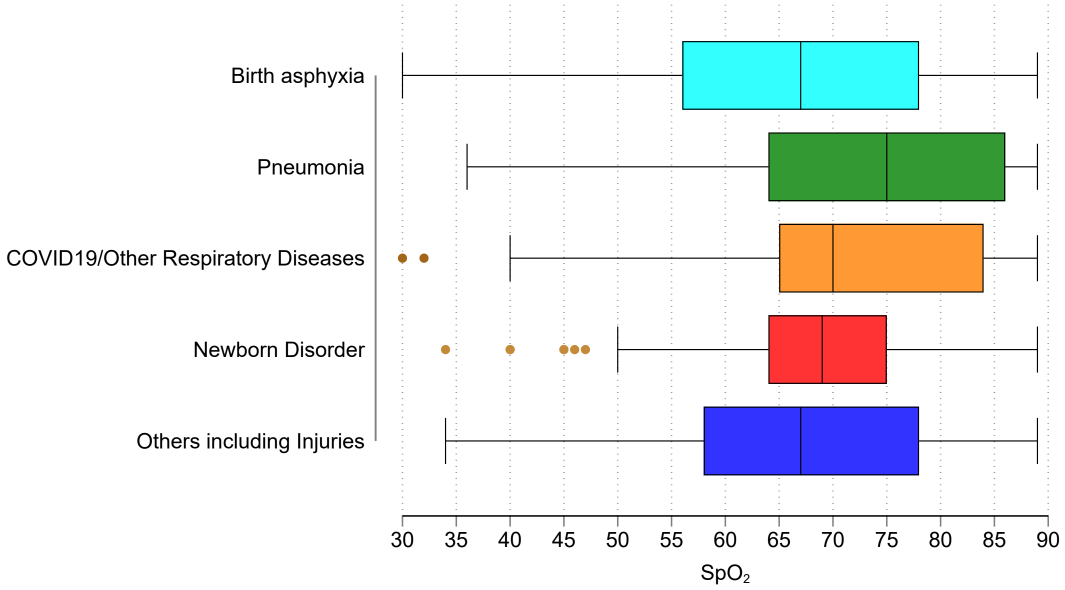

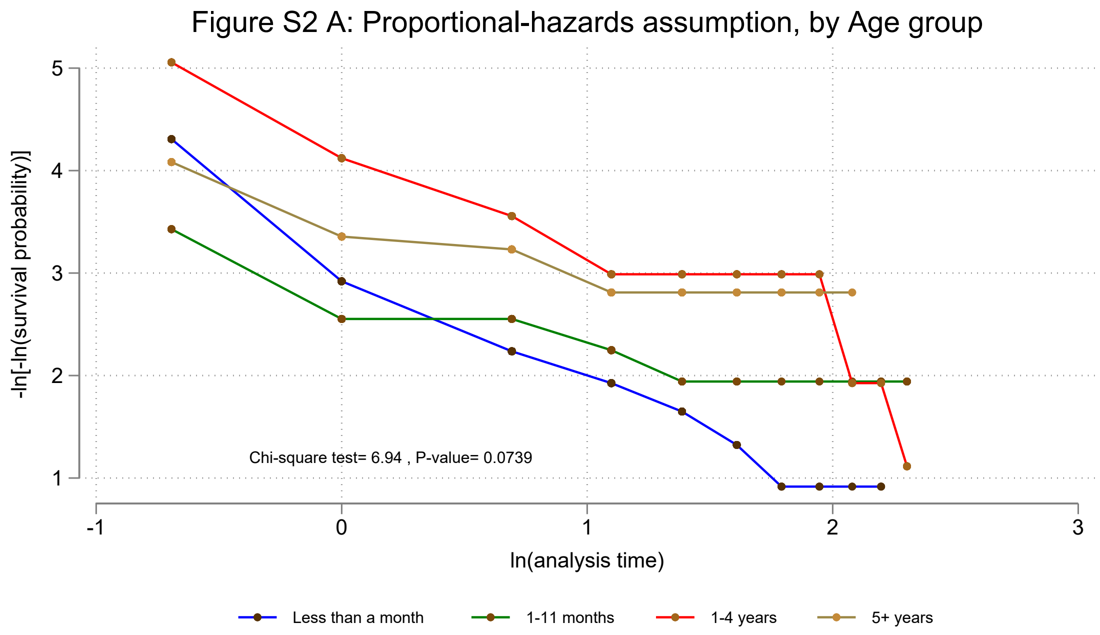

Figure S2 A: Proportional-hazards assumption, by Age group
-ln[-ln(survival probability)]
5
4
3
2
1
-1
0
1
2
3
ln(analysis time)
Chi-square test= 6.94 , P-value= 0.0739
Less than a month
1-11 months
1-4 years
5+ years

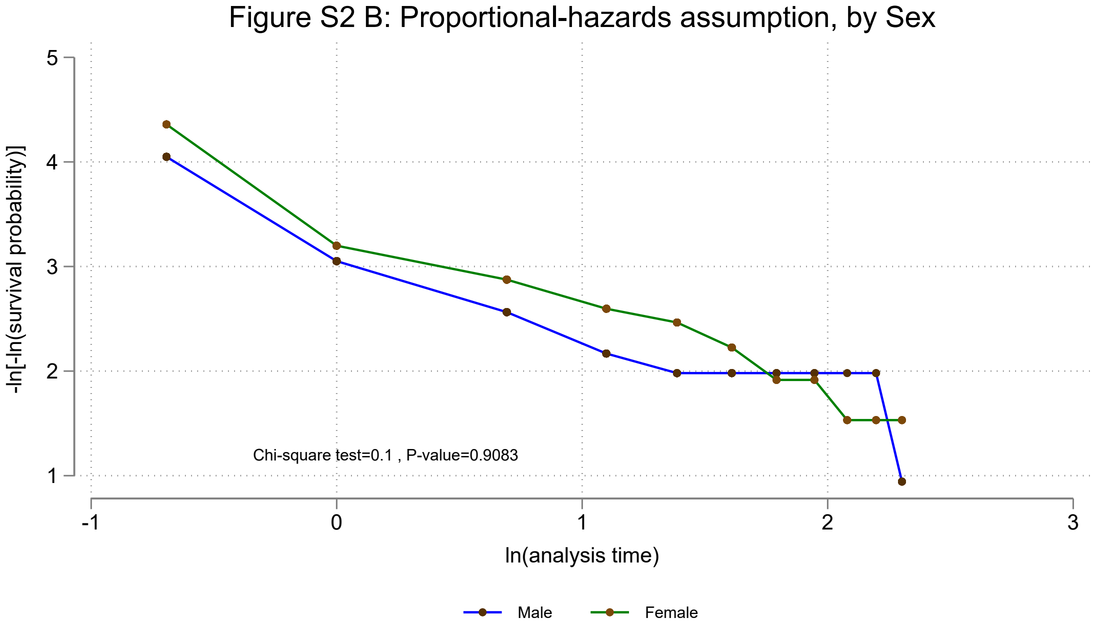
Figure S2 B: Proportional-hazards assumption, by Sex
-ln[-ln(survival probability)]
5
4
3
2
1
-1
0
1
2
3
ln(analysis time)
Chi-square test=0.1 , P-value=0.9083
Male
Female

Figure S2 C: Proportional-hazards assumption, by Indication for Oxygen therapy

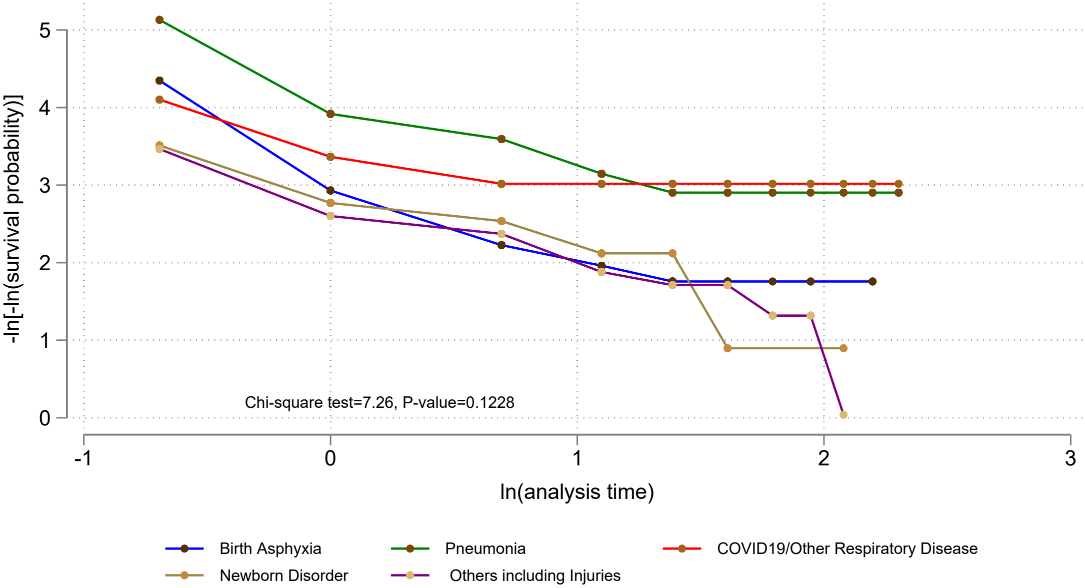

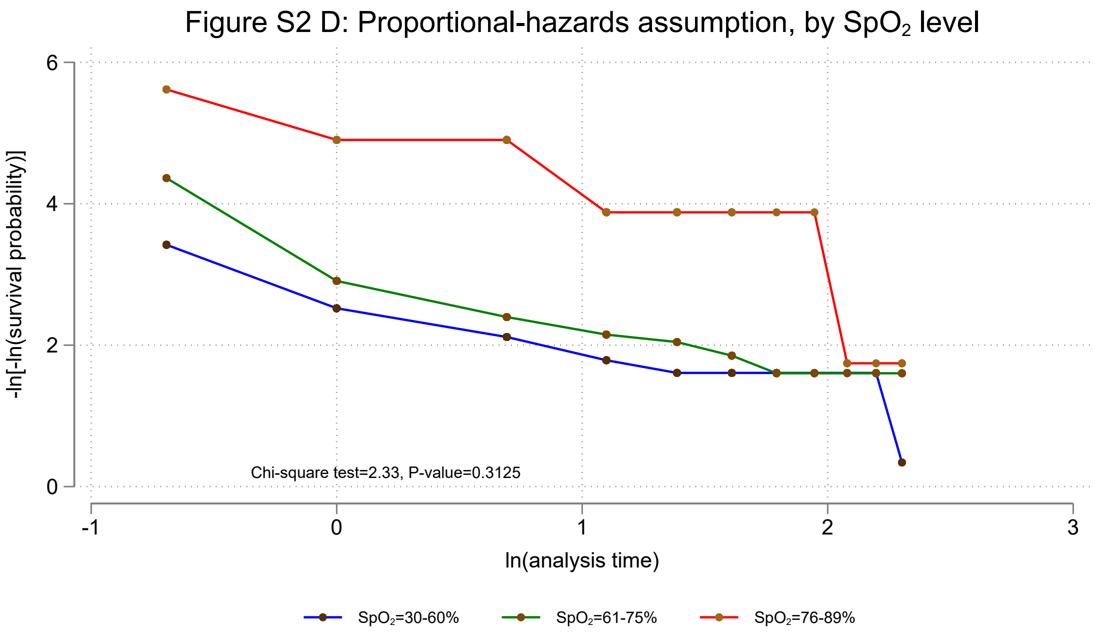

Figure S2 D: Proportional-hazards assumption, by SpO₂ level
-ln[-ln(survival probability)]
6
4
2
0
-1
0
1
2
3
ln(analysis time)
Chi-square test=2.33, P-value=0.3125
SpO₂=30-60%
SpO₂=61-75%
SpO₂=76-89%

Table S5. Crude hazard ratios of death during first 24 hours, after first day and the entire period

Variable/Characteristics	First 24 hours HR	First 24 hours 95%CI	First 24 hours *P-value*	After the first day HR	After the first day 95%CI	After the first day *P-value*	Entire period HR	Entire period 95%CI	Entire period *P-value*
**Age group**									
Less than a month	1.00			1.00			1.00		
1-11 months	1.04	(0.55 1.98)	*0.895*	**0.39**	**(0.19 0.77)**	*0.006*	**0.55**	**(0.31 0.96)**	*0.036*
1-4 years	**0.29**	**(0.11 0.77)**	*0.013*	**0.29**	**(0.14 0.58)**	*<0.001*	**0.32**	**(0.17 0.59)**	*<0.001*
5+years	1.20	(0.60 2.38)	*0.608*	0.53	(0.26 1.07)	*0.075*	0.73	(0.41 1.30)	*0.288*
Unobserved heterogeneity: thetah (SE)	2.78	(2.23)		0.98	(0.58)		1.09	(0.63)	
LR test of theta=0:	80.66			77.11			113.53		
**Sex**									
Female	1.00			1.00			1.00		
Male	0.89	(0.54 - 1.47)	*0.644*	0.81	(0.52 - 1.26)	*0.357*	0.80	(0.54 - 1.19)	*0.273*
Unobserved heterogeneity: thetah (SE)	3.14	(2.47)		1.16	(0.67)		1.24	(0.70)	
LR test of theta=0:	82.54			81.80			113.81		
**Reason for admission**									
Pneumonia	1.00			1.00			1.00		
Birth asphyxia	**3.52**	**(1.67 - 7.42)**	*0.001*	**5.07**	**(2.64 - 9.73)**	*<0.001*	**4.63**	**(2.58 - 8.31)**	*<0.001*
COVID19/Other Respiratory Diseases	2.99	(0.93 - 9.64)	*0.067*	1.44	(0.39 - 5.27)	*0.583*	2.14	(0.76 - 5.99)	*0.149*
Newborn Disorder	**3.62**	**(1.26 - 10.44)**	*0.017*	**3.18**	**(1.23 - 8.18)**	*0.017*	**3.97**	**(1.76 - 8.96)**	*0.001*
Others including Injuries	**5.80**	**(2.67 - 12.60)**	*<0.001*	**5.07**	**(2.55 - 10.06)**	*<0.001*	**5.65**	**(3.08 - 10.34)**	*<0.001*
Unobserved heterogeneity: thetah (SE)	**3.02**	(2.38)		**1.11**	(0.64)		**1.19**	(0.67)	
LR test of theta=0:	92.13			90.28			126.52		
**Oxygen saturation level on admission (SpO^2)**	**0.91**	**(0.89 - 0.92)**	*<0.001*	**0.91**	**(0.89 - 0.92)**	*<0.001*	**0.91**	**(0.89 - 0.92)**	*<0.001*
Unobserved heterogeneity: thetah (SE)	4.98	(3.53)		2.12	(1.07)		2.23	(1.11)	
LR test of theta=0:	175.90			183.85			255.13		

HR: Hazard Ratio, HR=1.00: Reference category, 95% CI: 95% Confidence Interval.

Reference

1. World Health Organization 2025 data.who.int, Somalia [Country overview]. Available at https://data.who.int/countries/706(Accessed on 17 November 2025).
2. WHO presence in fragile and conflict affected situations (FCS). Geneva: World Health Organization; 2021. https://iris.who.int/handle/10665/347261 (accessed November 12, 2025)
3. Breaking the gridlock: reimagining cooperation in a polarized world. New York: UNDP; 2024. Available at: https://hdr.undp.org/content/human-development-report-2023-24 (accessed November 17, 2024)
4. Somalia poverty and equity assessment. Washington, DC: World Bank Group; 2024. http://documents.worldbank.org/curated/en/099091824030521946/P500465195a18d08518a8c166461ccd0d10 (accessed December 25, 2024)
5. World Health Organization and the International Bank for Reconstruction and Development. Tracking universal health coverage: 2023 global monitoring report. Geneva: World Health Organization; 2023. https://iris.who.int/handle/10665/374059 (accessed Nov 7, 2024).
6. Nyagah LM, Bangura S, Omar OA, et al. The importance of community health workers as frontline responders during the COVID-19 pandemic, Somalia, 2020–2021. *Front Public Health*, 2023; **11**: 1215620. Available at: https://doi.org/10.3389/fpubh.2023.1215620
7. Somalia Harmonised Health Facility Assessment Report. National Report. October 2024. Federal Ministry of Health and Human Services, Mogadishu, Somalia. Available at https://moh.gov.so/so/pdfs/national-report-somalia-harmonised-health-facility-assessment-2022-2023/ (accessed November 17, 2025)
8. Country Cooperation Strategy for WHO and Somalia 2021–2025. Cairo: WHO Regional Office for the Eastern Mediterranean. 2022. Available at https://applications.emro.who.int/docs/9789290211280-eng.pdf?_gl=1*1gex9dv*_ga*MTY4NTg1OTk1MS4xNzI1MTc3NDAy*_ga_610FGB0GNK*czE3NjM0NTUxMjAkbzg3JGcxJHQxNzYzNDU1MTI3JGo1MyRsMCRoMA.. (accessed November 17, 2025)
9. Ibrahim AM, Mohamed MO, Osman MM, Mohamed LM, Hussein SA, Jama A. A gender-transformative approach towards reducing maternal mortality in Somalia. *The Lancet Obstetrics, Gynaecology, & Women's Health* 2025; **1**: e16.
10. Increasing access to medical oxygen in Somalia: fostering a resilient health system in fragile context. Case study. Mogadishu: World Health Organization Somalia; 2023. https://www.emro.who.int/images/stories/somalia/strengthening-public-health-systems-case-study-august-2-2023.pdf?ua=1 (accessed Nov 7, 2025).
